# Respiratory microbiome dysbiosis in STAT3 hyper-IgE syndrome (Job syndrome)

**DOI:** 10.1186/s13023-026-04508-x

**Published:** 2026-07-22

**Authors:** Cristian Roca, Matthew A. Greenwald, Bryan T. Zorn, Ling Sun, Gang Chen, Alexandra F. Freeman, Richard C. Boucher, Kenneth N. Olivier, Matthew C. Wolfgang

**Affiliations:** 1 Marsico Lung Institute/Cystic Fibrosis Research Center, University of North Carolina at Chapel Hill, Chapel Hill, NC USA; 2 Division of Pediatric Pulmonology, Department of Pediatrics, University of North Carolina at Chapel Hill, Chapel Hill, NC USA; 3 National Institutes of Allergy and Infectious Diseases, National Institutes of Health, Bethesda, MD USA; 4 Department of Microbiology and Immunology, University of North Carolina at Chapel Hill, 125 Mason Farm Road, 7105 Marsico Hall, Chapel Hill, NC 27599 USA

**Keywords:** STAT3, Hyper-IgE syndrome, Job syndrome, Microbiome, Rothia, Airway

## Abstract

STAT3 hyper-IgE syndrome (STAT3-HIES), historically known as Job syndrome, is a rare primary immunodeficiency caused by dominant-negative mutations in STAT3. STAT3-HIES is characterized by impaired mucosal immunity, connective tissue abnormalities, and recurrent cutaneous and pulmonary infections. Although STAT3-dependent immune and epithelial pathways are central to airway host defense, the respiratory microbiome in STAT3-HIES remains poorly defined. To address this question, we profiled upper and lower respiratory tract microbiomes using 16S rDNA gene sequencing of oral rinse samples from eight STAT3-HIES participants and eight matched healthy controls, and bronchoalveolar lavage fluid (BALF) samples from eight STAT3-HIES participants and six healthy controls that passed sequencing quality thresholds. Oral rinse samples from STAT3-HIES participants showed lower phylogenetic diversity and significantly altered microbial community structure compared with controls. In BALF, α-diversity was comparable between groups, but β-diversity differed significantly, indicating altered lower airway microbial community composition in STAT3-HIES. Differential abundance analysis identified enrichment of the genus Rothia in STAT3-HIES BALF, and Rothia relative abundance positively correlated with BALF IL-1β, IL-1RA, and IL-8. Although limited by cohort size, these findings provide the first characterization of respiratory microbiome alterations in STAT3-HIES and suggest that impaired STAT3-dependent immune pathways may influence airway microbial ecology and inflammatory responses.

## Introduction

Signal Transducer and Activator of Transcription 3 (STAT3) is a multifunctional transcription factor that integrates cytokine and growth factor signaling through the canonical Janus kinase (JAK)-STAT pathway [1–3]. STAT3 regulates diverse biological processes including cell proliferation, differentiation, immune signaling, and tissue development [4]. Proper STAT3 signaling is critical for host defense against bacterial and fungal pathogens at mucosal surfaces [5]. Dysfunctional STAT3 signaling has been implicated in numerous inflammatory diseases and immunodeficiencies [6].

Dominant-negative mutations in STAT3 cause STAT3 hyper-IgE syndrome (STAT3-HIES), a rare primary immunodeficiency characterized by markedly elevated serum IgE levels (> 2000 IU/mL), eczema, recurrent infections, and connective tissue abnormalities [7–11]. A defining immunologic feature of STAT3-HIES is impaired differentiation of Th17 lymphocytes, which is essential for epithelial barrier immunity [12–15]. Consequently, individuals with STAT3-HIES are particularly susceptible to infections caused by pathogens such as *Staphylococcus aureus* and *Candida* species.

Pulmonary disease is common in STAT3-HIES, with approximately 90% of affected individuals experiencing recurrent pneumonia [10, 16]. Repeated infections often lead to structural lung damage including bronchiectasis and pneumatoceles. Recent work has also demonstrated that STAT3 mutations compromise airway epithelial innate defenses beyond the well-described immune-cell defects [17]. Sun et al. show that STAT3 dysfunction reduces CFTR mRNA and protein expression, impairs CFTR-dependent fluid and mucin secretion, acidifies airway surface liquid, and diminishes antimicrobial peptide and cytokine/chemokine expression, collectively leading to defective mucociliary clearance and impaired bactericidal activity [17]. Critically, their clinical sputum analyses reveal that STAT3-HIES airways resemble pathophysiologic features observed in other muco-obstructive airway diseases such as cystic fibrosis and bronchiectasis, with STAT3-HIES sputum showing increased mucus concentration and heightened viscoelasticity, consistent with thick, tenacious secretions that are difficult to clear [17]. In muco-obstructive airway diseases, airway microbial communities play a central role in disease progression [18–20].

STAT3-HIES structural abnormalities, combined with impaired immune signaling, may alter the airway microbial environment and promote persistent colonization by opportunistic microbes such as *Pseudomonas aeruginosa*, nontuberculous mycobacteria, and *Aspergillus* species [21, 22]. Despite these clinical observations, the respiratory microbiome of STAT3-HIES has not been systematically characterized, and to our knowledge no prior studies have directly examined microbial community structure in the lower airways of STAT3-HIES individuals.

Since prior microbiome studies in STAT3-HIES have focused largely on the skin and oral mucosa, little is known about microbial community dynamics in the respiratory tract [23–26]. Here we performed microbiome analysis using samples from a previously published cohort [17] to characterize oral and lower airway microbiomes in STAT3-HIES. Given the broad role of STAT3 in epithelial and immune regulation, we hypothesized that STAT3-HIES would be associated with distinct microbial community structures in both the upper and lower respiratory tract.

## Methods

### Sample population and collection

This report utilizes specimens collected and archived from a previously published study cohort [17]. Respiratory tract specimens were collected with informed consent under IRB protocol #07-H-0142 at the National Heart, Lung, and Blood Institute (Bethesda, MD). Healthy volunteers needed to be between 18 and 75 years old in good general health without clinically significant medical history, physical exam findings or screening lab and chest x-ray abnormalities with ability to comply with requirements for conscious sedation and willingness to donate specimens for storage and future research. Individuals with a smoking history of 10 pack-years or more, current smokers, or tobacco free for less than a year were excluded. Upper respiratory oral rinse (OR) and lower respiratory bronchoalveolar lavage fluid (BALF) samples were collected from eight STAT3-HIES participants and eight age- and sex-matched healthy controls. Subjects were not to eat or drink anything for six hours prior and two hours after the procedure. The bronchopulmonary segment for BALF collection was selected according to predefined criteria, with preference given to the middle lobe or lingula. BALF was collected by instilling 50 mL of sterile saline into the bronchopulmonary segment via bronchoscope, followed by gentle suction aspiration. Oral rinse samples were collected by instructing participants to gargle sterile saline for 30 s before expelling the rinse into sterile collection tubes. Samples were immediately frozen at − 80°C until analysis.

### Microbiome sequencing

DNA was extracted from frozen oral rinse and BALF samples using the DNeasy PowerSoil kit (Qiagen). Two samples (negative controls) with molecular grade water underwent DNA extraction and sequencing at the same time as the study samples using the same techniques. Negative controls were used to decontaminate the study samples using the prevalence method of decontamination [27]. Bacterial DNA was amplified using the Quick-16S library preparation kit (Zymo Research), targeting the V4 region of the bacterial 16S rRNA gene. Sequencing the V4 region alone has been shown to more closely resemble full-length 16S taxonomic assignment compared to V3-V4, in a phenotypically similar rare lung disease [28]. Intermediate region between V3-V4 regions may be highly variable and drive Phred scores (quality scores) lower than V4 alone. Libraries were sequenced on an Illumina NextSeq2000 platform with paired-end reads. Sequencing quality was assessed using FastQC prior to downstream analysis. Two samples (two healthy control BALF samples) were excluded due to low-quality scores.

### Bioinformatic processing and statistical analysis

Sequence reads were mapped to the Human Oral Microbiome Database (HOMD) [29] and processed using the DADA2-QIIME2 pipeline to generate amplicon sequence variant (ASV) tables. Taxonomic assignments were integrated with sample metadata using the Phyloseq package in R (v4.4.1). Rarefaction was performed at 5,000 reads. α-diversity was assessed using Shannon and Faith’s phylogenetic diversity metrics. Group comparisons were performed using the Mann–Whitney U test. β-diversity was evaluated using UniFrac distance and PERMANOVA. Microbiome differential abundance analysis was performed using Analysis of Composition of Microbiome with Bias Correction (ANCOM-BC2) [30], to compare taxa across groups while correcting for both sample-specific and taxon-specific bias.

### Cytokine and chemokine measurements

Cytokine and chemokine measurements were obtained from the original Sun et al. dataset [17]. BALF cytokines were quantified using a U-PLEX multiplex immunoassay (Meso Scale Diagnostics). In brief, 96-well plates were coated with linker-coupled capture antibodies for one hour, followed by three washes with PBS with 0.05% Tween-20 (PBST). 25µL of standards and samples were added and incubated for one hour at room temperature with shaking. Plates were then washed three times, incubated for one hour with detection antibodies then followed by another three washes. After adding 150µL of reading buffer per well, plates were immediately read on a MESO QUICKPLEX SQ 120 instrument using an electrochemiluminescent detection method. DISCOVERY WORKBENCH software (Meso Scale Diagnostics) was used for sample analysis. Standard curves were generated using a weighted four-parameter logistic model fit. Correlations between microbial taxa and cytokine levels were evaluated using Spearman correlation analysis in GraphPad Prism.

## Results

### Study cohort

Upper respiratory oral rinse and lower respiratory BALF samples were obtained from eight STAT3-HIES participants and eight matched healthy controls. All STAT3-HIES participants were clinically stable at the time of sampling, although several exhibited chronic pulmonary complications including bronchiectasis and pneumatoceles (Table 1). All STAT3-HIES participants were maintained on their chronic antimicrobial regimens. Because discontinuing therapy solely for research purposes was not ethically permissible given the risk of pulmonary deterioration, antimicrobial exposure represents an important study limitation and potential confounding variable.

### Upper airway microbiome

**Fig. 1:**
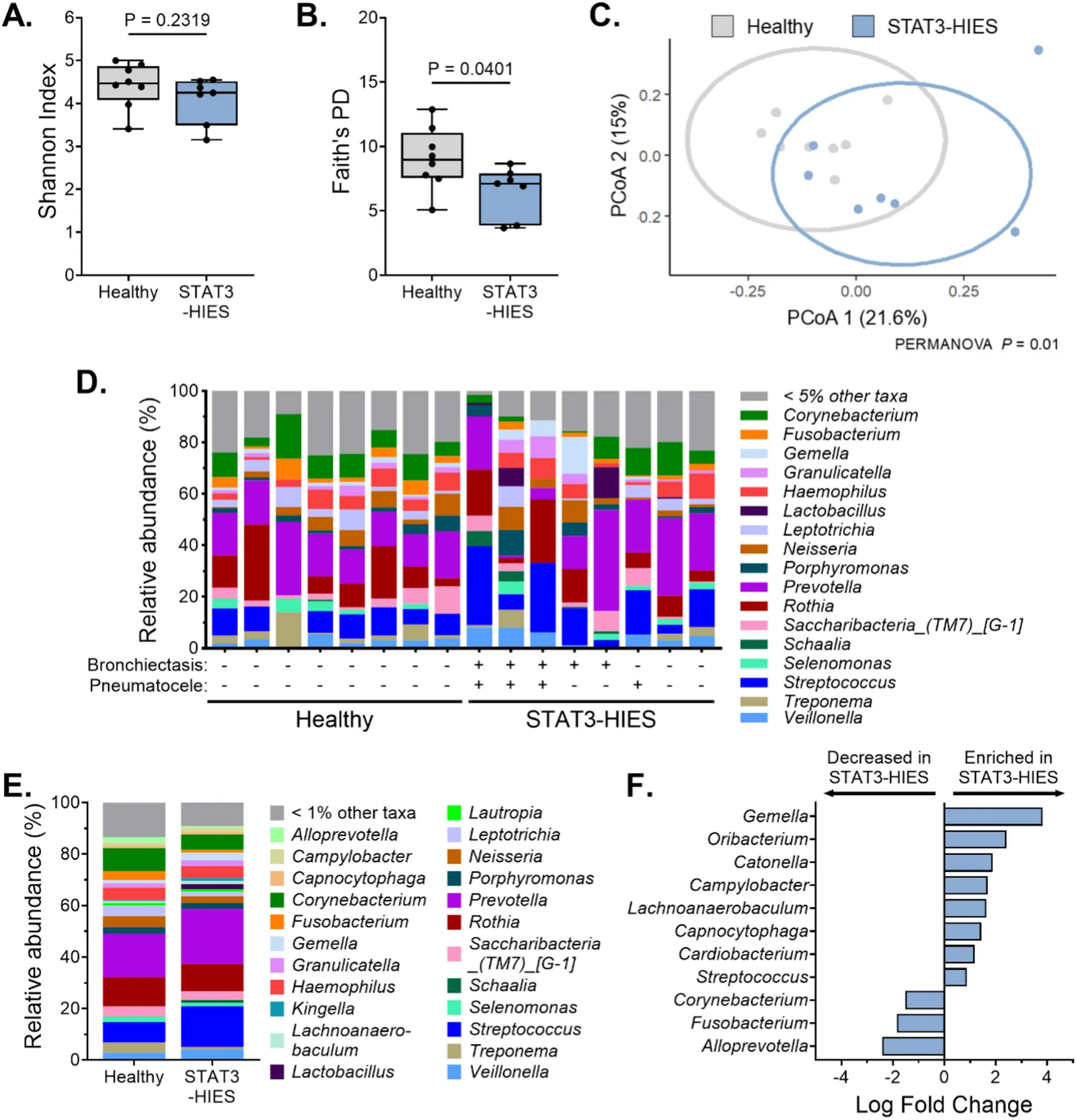
Oral microbial dysbiosis in STAT3-HIES**.** Microbiome analysis of oral rinse (OR) samples from STAT3-HIES (*n* = 8) and healthy controls (*n* = 8). **A&B)** Indices of sample α-diversity by group; **(A)** Shannon index and **(B)** Faith's phylogenetic diversity (PD). **(C)** β-diversity principal coordinate analysis (PCoA) by Unifrac distance with accompanying PERMANOVA statistical test. **D&E)** Bacterial relative abundance by **(D)** individual participant and **(E)** disease group. **(F)** Analysis of composition of microbiome with bias correction (ANCOM-BC2) demonstrating differential abundance of bacterial genera

Analysis of oral rinse samples revealed a trend toward reduced bacterial α-diversity in STAT3-HIES participants compared with healthy controls, as measured by both Shannon diversity (richness and evenness) and Faith’s phylogenetic diversity (total branch length represented). (Fig. 1A-B). This suggests that STAT3-HIES oral communities are not only less taxonomically diverse but also occupy a narrower span of the bacterial phylogenetic tree. β-diversity analysis demonstrated significant separation between groups (Fig. 1C; PERMANOVA *P* = 0.01), indicating shifts in overall community composition between groups based on between-sample dissimilarity metrics. Despite these community-level differences, substantial inter-individual variability limited detection of statistically significant genus-level differences (*q* > 0.1) (Fig. 1D-E). The most notable shift was a non-significant increase in the relative abundance of the genus *Gemella* in STAT3-HIES samples (Fig. 1F).

### Lower airway microbiome

**Fig. 2:**
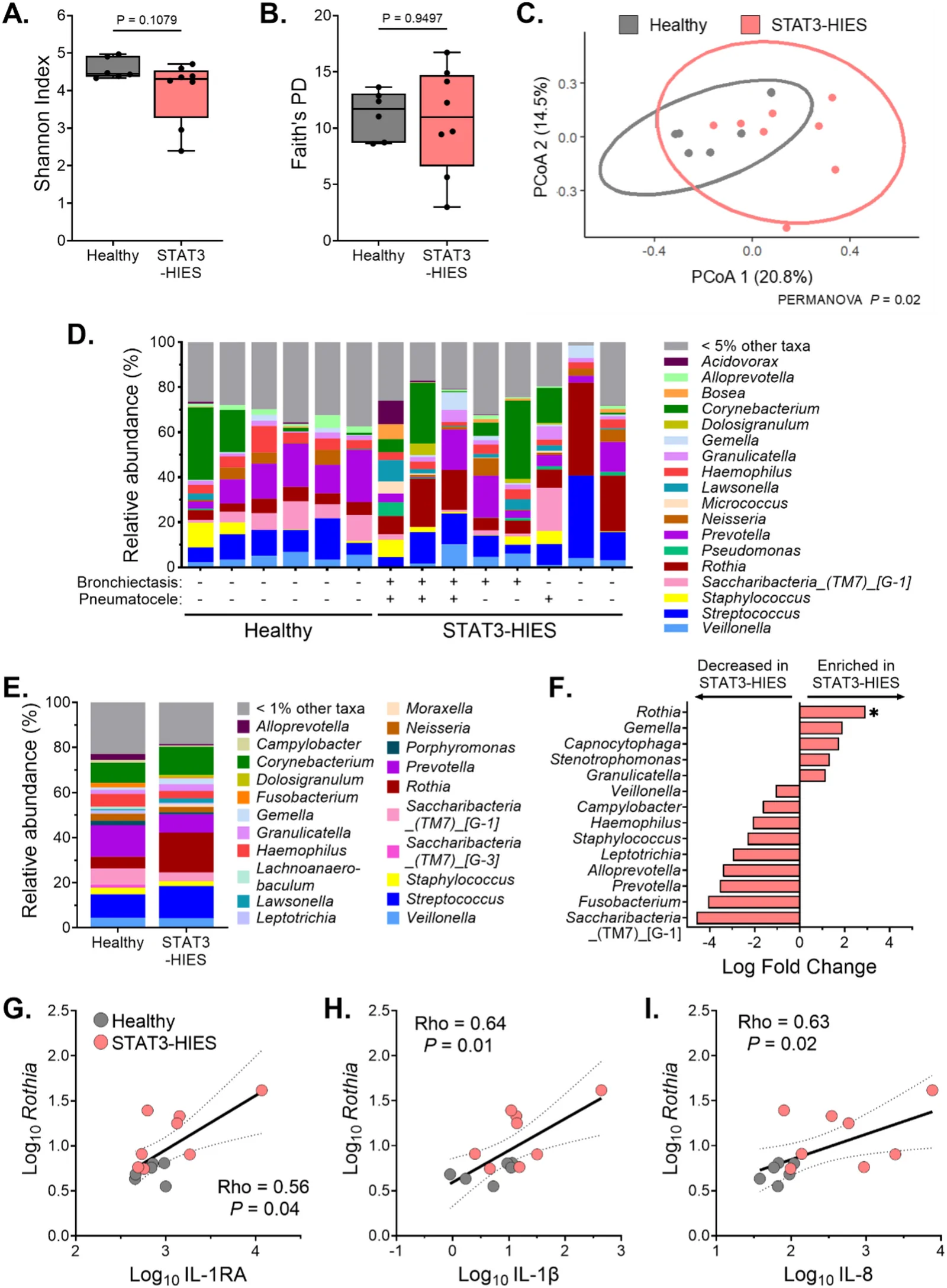
Taxonomic differences in lower airway BALF samples demonstrate that enrichment of *Rothia *is correlated with airway inflammation. **A&B)** α-diversity metrics by group (*n*=6 healthy control and *n*=8 STAT3-HIES). **(C)** Unifrac PCoA comparison with PERMANOVA statistical analysis. **D&E)** Relative abundance of bacteria at the genus taxonomic level. F) ANCOM-BC2 differential taxonomic analysis with bias correction. *P<0.05 **G-I)** Spearman correlation between relative abundance of *Rothia *in BALF and inflammatory cytokines **(G)** IL-1RA, **(H)** IL-1β, and **(I)** IL-8

α-diversity of BALF samples was comparable between STAT3-HIES participants and controls (Fig. 2A-B). However, β-diversity analysis revealed significant differences in microbial community composition between groups (Fig. 2C; PERMANOVA *P* = 0.02). This result implies that STAT3-HIES and controls harbor a similar number of taxa and comparable within-sample richness/evenness (Fig. 2A-B), but composition of those communities differs between groups (i.e., relative abundance of taxa present) (Fig. 2C). BALF samples contained low levels of opportunistic respiratory pathogens including *Haemophilus*, *Pseudomonas*, *Stenotrophomonas*, and *Staphylococcus*, which were not detected in corresponding oral rinse samples (Figs. 1D-E and 2D-E). Compared to healthy controls, STAT3-HIES participants showed lower relative abundance of several putative respiratory commensals including *Prevotella*, *Fusobacterium*, and Saccharibacteria TM7 G1 and a significant enrichment of the genus *Rothia* (Fig. 2F).

### Association between *Rothia* and airway inflammation

Correlation analysis demonstrated that relative abundance of *Rothia* positively correlated with BALF levels of IL-1β, IL-8, and IL-1RA across the combined cohort (Fig. 2G–I). However, *Rothia* abundance did not correlate with clinical disease severity, including ppFEV_1_ and the presence of bronchiectasis or pneumatoceles (Table 1).

## Discussion

This study provides an initial characterization of respiratory microbiome alterations in individuals with STAT3-HIES. Despite the small cohort size, we identified differences in both microbial diversity and community composition associated with STAT3-HIES. The most notable finding was enrichment of *Rothia* in the lower airways of STAT3-HIES participants, which correlated with inflammatory cytokine levels.

The oral microbiome of STAT3-HIES individuals exhibited reduced phylogenetic diversity and significantly divergent community structure (β-diversity) compared to healthy controls. These findings are consistent with previous reports demonstrating immune dysregulation in the oral mucosa and oral microbial dysbiosis in STAT3-HIES [31]. Oral dysbiosis is a hallmark for the development of dental caries [31, 32] and may explain the increased incidence of caries in STAT3-HIES compared to the general population [33].

In contrast, the lower airway microbiome showed preserved overall diversity but distinct taxonomic composition. A major contributor to this divergence was the enrichment of *Rothia* in BALF samples. Interestingly, this enrichment was not observed in matched oral rinse samples, suggesting that expansion of *Rothia* may reflect local immune and epithelial conditions associated with STAT3-HIES rather than simple upper airway contamination.

One potential explanation for these microbial shifts is the central role of STAT3 in Th17-mediated mucosal immunity. STAT3 signaling is required for differentiation of Th17 cells affecting the production and signaling of IL-17 and IL-22, cytokines that regulate epithelial antimicrobial peptide expression, neutrophil recruitment, and mucosal barrier integrity [13–15, 34]. Impaired Th17 responses in STAT3-HIES may therefore alter the selective pressures that normally shape airway microbial communities, allowing expansion of organisms that are otherwise minor members of the respiratory microbiome.

*Rothia* species are Gram-positive facultative anaerobic, encapsulated bacteria, commonly found in the oral cavity and respiratory tract [35, 36]. In our cohort, the relatively abundance of *Rothia* in the lower airways of STAT3-HIES correlated positively with IL-1β, IL-8, and IL-1RA in BALF. Moreover, when these cytokine and chemokines were analyzed by disease, only IL-1β reached statistical significance between groups, with IL-1β elevated in STAT3-HIES [17]. These cytokines are key mediators of innate immune responses and neutrophil recruitment, suggesting that *Rothia* may contribute to or be shaped by local inflammatory signaling in the lungs.

*Rothia* species are emerging as influential members of the lung microbiome in individuals with chronic muco-obstructive diseases and have been reported to exert both anti-inflammatory and pro-inflammatory effects, depending on host context and immune status [37, 38]. In addition to immune signaling defects, dominant-negative STAT3 mutations also disrupt epithelial host defense pathways that maintain airway microbial homeostasis. Impaired STAT3-dependent signaling in STAT3-HIES has been shown to alter mucociliary clearance and airway surface liquid regulation through defects in ciliogenesis, reduced CFTR expression, and altered mucin production [17]. These epithelial abnormalities may further influence microbial community structure by altering airway surface liquid composition and mucociliary transport, creating ecological niches that favor colonization by specific bacterial taxa [17]. However, mechanisms by which *Rothia* contributes to or responds to disease pathology remain enigmatic. *Rothia mucilaginosa* and *Rothia aeria* have been linked to pneumonia in both immunocompromised individuals and immunocompetent individuals with chronic lung diseases or structural airway abnormalities like bronchiectasis [39, 40]. However, other studies suggest *Rothia* can attenuate inflammatory signaling, in part by dampening activation of the NF-κB pathway [37, 41, 42]. Ultimately, these reports highlight the need to delineate species- and strain-specific functions of *Rothia* within the lung microbiome, particularly in the context of disease specific immune dysregulation.

This study has several limitations, including potential for oral contamination during bronchoscopy, sequencing only the 16S V4 region, confounding antibiotic exposure in the STAT3-HIES group, and most notably a small cohort size (*n* = 6–8). The pronounced interindividual variability further limited the ability to draw robust conclusions. Given the small sample size and subject variability, the overall outcomes drawn may be sensitive to minor changes in the dataset. However, because *Rothia* enrichment was observed in BALF, but not in matched oral rinse samples, this finding may reflect microbial community differences within the lower airways rather than simple carryover from the upper airway. Future studies using metagenomic sequencing and larger multicenter cohorts, with additional disease control groups, such as individuals with cystic fibrosis or primary immunodeficiencies, who also receive chronic antibiotic therapy and could help disentangle antibiotic-specific effects, will be required to define species-level microbial signatures and determine whether airway microbiome alterations contribute to pulmonary disease progression in STAT3-HIES.

**Table 1:** Demographic and clinical characteristics of the study population

Subject	Group	Age	Sex	Race	ppFEV_1_	Additional Diagnosis by chest CT	STAT3 Mutation^#^	Antimicrobial Regimen
1	STAT3-HIES	27	M	Caucasian/white	62	bronchiectasis + pneumatocele	1027 G > T; V343F	azithromycin, posaconazole, levofloxacin
2	STAT3-HIES	28	M	Caucasian/white	55	bronchiectasis + pneumatocele	1997 T > G; L666R	TMP/SMX, posaconazole
3	STAT3-HIES	44	F	Caucasian/white	52	bronchiectasis	1144 C > T; R382W	posaconazole
4	STAT3-HIES	32	M	African American/ Black	42	bronchiectasis + pneumatocele	1144 C > T; R382W	azithromycin, TMP/SMX, levofloxacin, posaconazole
5	STAT3-HIES	21	F	Caucasian/white	71	pneumatoceles	1387delGTG; V463del	TMP/SMX, posaconazole
6	STAT3-HIES	40	F	Caucasian/white	78	bronchiectasis	1144 C > T; R382W	cefepime, clindamycin, daptomycin, caspofungin,
7	STAT3-HIES	25	F	Asian	68	Normal chest CT	1393 T > C; S465P	TMP/SMX, fluconazole
8	STAT3-HIES	21	F	Caucasian/white	88	Normal chest CT	1145 G > A; R382Q	doxycycline, posaconazole
9	Healthy control	37	F	African American/ Black	-	-	-	
10	Healthy control	23	M	Caucasian/white	-	-	-	
11	Healthy control	40	F	African American/ Black	-	-	-	
12	Healthy control	36	M	Caucasian/white	-	-	-	
13	Healthy control	43	M	Hawaiian/ Pacific Islander	-	-	-	
14	Healthy control	23	M	African American/ Black	-	-	-	
15	Healthy control	22	F	Unknown	-	-	-	
16	Healthy control	22	F	Unknown	-	-	-	

^#^ Genotype change; Amino acid change ppFEV_1_ = percent predicted forced expiratory volume in 1 second STAT3-HIES = Signal Transducer and Activator of Transcription 3 Hyper-Immunoglobulin E Syndrome CT = Computed Tomography TMP/SMX = Trimethoprim-Sulfamethoxazole

## Data Availability

The sequencing datasets generated and analyzed in this study are available in the Sequence Read Archive under Bioproject ID PRJNA1447540.

## Abbreviations

ANCOM-BC2: Analysis of composition of microbiome with bias correction
ASV: Amplicon sequence variant
BALF: Bronchoalveolar lavage fluid
CFTR: Cystic fibrosis transmembrane conductance regulator
CT: Computed tomography
HIES: Hyper-immunoglobulin E syndrome
HOMD: Human oral microbiome database
IgE: Immunoglobulin E
IL: Interleukin
IRB: Institutional review board
JAK: Janus kinase
NHLBI: National heart, lung, and blood institute
NIH: National institutes of health
OR: Oral rinse
PCoA: Principal coordinate analysis
PD: Phylogenetic diversity
STAT3: Signal transducer and activator of transcription 3

## References

[1] Ihle JN, Kerr IM. Jaks and Stats in signaling by the cytokine receptor superfamily. Trends Genet. 1995;11:69–74. 10.1016/s0168-9525(00)89000-9 7716810

[2] Levy DE, Lee C. What does Stat3 do? J Clin Invest. 2002;109:1143–8. 10.1172/JCI15650 PMC150972 11994402

[3] Samad MA, Ahmad I, Hasan A, Alhashmi MH, Ayub A, Al-Abbasi FA, et al. STAT3 signaling pathway in health and disease. MedComm. 2025;6:e70152. 10.1002/mco2.70152 PMC11955304 40166646

[4] O’Shea JJ, Holland SM, Staudt LM. JAKs and STATs in immunity, immunodeficiency, and cancer. N Engl J Med. 2013;368:161–70. 10.1056/NEJMra1202117 PMC7604876 23301733

[5] Vinh DC, Sugui JA, Hsu AP, Freeman AF, Holland SM. Invasive fungal disease in autosomal-dominant hyper-IgE syndrome. J Allergy Clin Immunol. 2010;125:1389–90. 10.1016/j.jaci.2010.01.047 PMC2879472 20392475

[6] Hillmer EJ, Zhang H, Li HS, Watowich SS. STAT3 signaling in immunity. Cytokine Growth Factor Rev. 2016;31:1–15. 10.1016/j.cytogfr.2016.05.001 PMC5050093 27185365

[7] Minegishi Y, Saito M, Tsuchiya S, Tsuge I, Takada H, Hara T, et al. Dominant-negative mutations in the DNA-binding domain of STAT3 cause hyper-IgE syndrome. Nature. 2007;448:1058–62. 10.1038/nature06096 17676033

[8] Renner ED, Torgerson TR, Rylaarsdam S, Añover-Sombke S, Golob K, LaFlam T, et al. STAT3 mutation in the original patient with Job’s syndrome. N Engl J Med. 2007;357:1667–8. 10.1056/NEJMc076367 17942886

[9] Gernez Y, Freeman AF, Holland SM, Garabedian E, Patel NC, Puck JM, et al. Autosomal Dominant Hyper-IgE Syndrome in the USIDNET Registry. J Allergy Clin Immunol Pract. 2018;6:996–1001. 10.1016/j.jaip.2017.06.041 PMC5858974 28939137

[10] Al-Shaikhly T, Ochs HD. Hyper IgE syndromes: clinical and molecular characteristics. Immunol Cell Biol. 2019;97:368–79. 10.1111/imcb.12209 30264496

[11] Holland SM, DeLeo FR, Elloumi HZ, Hsu AP, Uzel G, Brodsky N, et al. STAT3 mutations in the hyper-IgE syndrome. N Engl J Med. 2007;357:1608–19. 10.1056/NEJMoa073687 17881745

[12] Foley JF. STAT3 regulates the generation of th17 cells. Science’s STKE. 2007;2007:tw113–113.

[13] Milner JD, Brenchley JM, Laurence A, Freeman AF, Hill BJ, Elias KM, et al. Impaired T(H)17 cell differentiation in subjects with autosomal dominant hyper-IgE syndrome. Nature. 2008;452:773–6. 10.1038/nature06764 PMC2864108 18337720

[14] Renner ED, Rylaarsdam S, Anover-Sombke S, Rack AL, Reichenbach J, Carey JC, et al. Novel signal transducer and activator of transcription 3 (STAT3) mutations, reduced T(H)17 cell numbers, and variably defective STAT3 phosphorylation in hyper-IgE syndrome. J Allergy Clin Immunol. 2008;122:181–7. 10.1016/j.jaci.2008.04.037 PMC4560358 18602572

[15] Zheng Y, Valdez PA, Danilenko DM, Hu Y, Sa SM, Gong Q, et al. Interleukin-22 mediates early host defense against attaching and effacing bacterial pathogens. Nat Med. 2008;14:282–9. 10.1038/nm1720 18264109

[16] Tsilifis C, Freeman AF, Gennery AR. STAT3 Hyper-IgE Syndrome-an Update and Unanswered Questions. J Clin Immunol. 2021;41:864–80. 10.1007/s10875-021-01051-1 PMC8249299 33932191

[17] Sun L, Walls SA, Dang H, Quinney NL, Sears PR, Sadritabrizi T, et al. STAT3-dependent regulation of CFTR and ciliogenesis is essential for mucociliary clearance and innate airway defense in Hyper-IgE syndrome. Am J Respir Crit Care Med. 2025;211(10):1951–69. 10.1164/rccm.202407-1415OC PMC12555045 40315437

[18] Natalini JG, Singh S, Segal LN. The dynamic lung microbiome in health and disease. Nat Rev Microbiol. 2023;21:222–35. 10.1038/s41579-022-00821-x PMC9668228 36385637

[19] Richardson H, Dicker AJ, Barclay H, Chalmers JD. The microbiome in bronchiectasis. Eur Respir Rev. 2019;28. 10.1183/16000617.0048-2019 PMC9489022 31484665

[20] Muhlebach MS, Zorn BT, Esther CR, Hatch JE, Murray CP, Turkovic L, et al. Initial acquisition and succession of the cystic fibrosis lung microbiome is associated with disease progression in infants and preschool children. PLoS Pathog. 2018;14:e1006798. 10.1371/journal.ppat.1006798 PMC5773228 29346420

[21] Zhang Y, Lin T, Leung HM, Zhang C, Wilson-Mifsud B, Feldman MB, et al. STAT3 mutation-associated airway epithelial defects in Job syndrome. J Allergy Clin Immunol. 2023;152:538–50. 10.1016/j.jaci.2022.12.821 PMC10330947 36638921

[22] Xie T, Xu N, Zhao H, Han Y, Wu J, Di H, et al. Pulmonary features and stage of disease in adult patients with hyper-IgE syndrome: a single-centre clinical study and literature review. Orphanet J Rare Dis. 2025;20:270. 10.1186/s13023-025-03749-6 PMC12131643 40462219

[23] Oh J, Freeman AF, Comparative Sequencing NISC, Program, Park M, Sokolic R, Candotti F, et al. The altered landscape of the human skin microbiome in patients with primary immunodeficiencies. Genome Res. 2013;23:2103–14. 10.1101/gr.159467.113 PMC3847779 24170601

[24] Smeekens SP, Huttenhower C, Riza A, van de Veerdonk FL, Zeeuwen PLJM, Schalkwijk J, et al. Skin microbiome imbalance in patients with STAT1/STAT3 defects impairs innate host defense responses. J Innate Immun. 2014;6:253–62. 10.1159/000351912 PMC4045018 23796786

[25] Sharma M, Dhaliwal M, Tyagi R, Goyal T, Sharma S, Rawat A. Microbiome and its dysbiosis in inborn errors of immunity. Pathogens. 2023;12. 10.3390/pathogens12040518 PMC10145396 37111404

[26] Guttman-Yassky E, Irvine AD, Brunner PM, Kim BS, Boguniewicz M, Parmentier J, et al. The role of Janus kinase signaling in the pathology of atopic dermatitis. J Allergy Clin Immunol. 2023;152:1394–404. 10.1016/j.jaci.2023.07.010 37536511

[27] Davis NM, Proctor DM, Holmes SP, Relman DA, Callahan BJ. Simple statistical identification and removal of contaminant sequences in marker-gene and metagenomics data. Microbiome. 2018;6:226. 10.1186/s40168-018-0605-2 PMC6298009 30558668

[28] Hahn A, Sanyal A, Perez GF, Colberg-Poley AM, Campos J, Rose MC, et al. Different next generation sequencing platforms produce different microbial profiles and diversity in cystic fibrosis sputum. J Microbiol Methods. 2016;130:95–9. 10.1016/j.mimet.2016.09.002 PMC5065784 27609714

[29] Chen T, Yu W-H, Izard J, Baranova OV, Lakshmanan A, Dewhirst FE. The Human Oral Microbiome Database: a web accessible resource for investigating oral microbe taxonomic and genomic information. Database (Oxford). 2010;2010:baq013. 10.1093/database/baq013 PMC2911848 20624719

[30] Lin H, Peddada SD. Multigroup analysis of compositions of microbiomes with covariate adjustments and repeated measures. Nat Methods. 2024;21:83–91. 10.1038/s41592-023-02092-7 PMC10776411 38158428

[31] Abusleme L, Diaz PI, Freeman AF, Greenwell-Wild T, Brenchley L, Desai JV et al. Human defects in STAT3 promote oral mucosal fungal and bacterial dysbiosis. JCI Insight. 2018;3. 10.1172/jci.insight.122061 PMC6171814 30185668

[32] Dinis M, Agnello M, Cen L, Shokeen B, He X, Shi W, et al. Oral Microbiome: Streptococcus mutans/Caries Concordant-Discordant Children. Front Microbiol. 2022;13:782825. 10.3389/fmicb.2022.782825 PMC8891984 35250921

[33] Esposito L, Poletti L, Maspero C, Porro A, Pietrogrande MC, Pavesi P, et al. Hyper-IgE syndrome: dental implications. Oral Surg Oral Med Oral Pathol Oral Radiol. 2012;114:147–53. 10.1016/j.oooo.2012.04.005 22769403

[34] Yang XO, Panopoulos AD, Nurieva R, Chang SH, Wang D, Watowich SS, et al. STAT3 regulates cytokine-mediated generation of inflammatory helper T cells. J Biol Chem. 2007;282:9358–63. 10.1074/jbc.C600321200 17277312

[35] Chaturvedi AK, Vogtmann E, Shi J, Yano Y, Blaser MJ, Bokulich NA, et al. Oral microbiome profile of the US population. JAMA Netw Open. 2025;8:e258283. 10.1001/jamanetworkopen.2025.8283 PMC12053784 40323603

[36] West SR, Suddaby AB, Lewin GR, Ibberson CB. Rothia Trends Microbiol. 2024;32:720–1. 10.1016/j.tim.2024.03.009 38580605

[37] Rigauts C, Aizawa J, Taylor SL, Rogers GB, Govaerts M, Cos P, et al. Rothia mucilaginosa is an anti-inflammatory bacterium in the respiratory tract of patients with chronic lung disease. Eur Respir J. 2022;59. 10.1183/13993003.01293-2021 PMC9068977 34588194

[38] Yen T-Y, Hsu C, Lee N-C, Wu C-S, Wang H, Lee K-Y, et al. Signatures of lower respiratory tract microbiome in children with severe community-acquired pneumonia using shotgun metagenomic sequencing. J Microbiol Immunol Infect. 2025;58:86–93. 10.1016/j.jmii.2024.11.011 39643526

[39] Maraki S, Papadakis IS. Rothia mucilaginosa pneumonia: a literature review. Infect Dis (Lond). 2015;47:125–9. 10.3109/00365548.2014.980843 25664502

[40] Sonehara K, Araki T, Hanaoka M. Rothia aeria pneumonia in an immunocompetent patient: A novel case study. Respirol Case Rep. 2021;9:e0843. 10.1002/rcr2.843 PMC8455964 34584726

[41] Gao B, Gallagher T, Zhang Y, Elbadawi-Sidhu M, Lai Z, Fiehn O, et al. Tracking polymicrobial metabolism in cystic fibrosis airways: Pseudomonas aeruginosa metabolism and physiology are influenced by Rothia mucilaginosa-derived metabolites. mSphere. 2018;3. 10.1128/mSphere.00151-18 PMC5917424 29695623

[42] Goeteyn E, Grassi L, Van den Bossche S, Rigauts C, Vande Weygaerde Y, Van Braeckel E, et al. Commensal bacteria of the lung microbiota synergistically inhibit inflammation in a three-dimensional epithelial cell model. Front Immunol. 2023;14:1176044. 10.3389/fimmu.2023.1176044 PMC10164748 37168857

